# Tunable on-chip mode converter enabled by inverse design

**DOI:** 10.1515/nanoph-2022-0638

**Published:** 2023-02-17

**Authors:** Hongyin Zhou, Kun Liao, Zhaoxian Su, Tianhao Li, Guangzhou Geng, Junjie Li, Yongtian Wang, Xiaoyong Hu, Lingling Huang

**Affiliations:** Beijing Engineering Research Center of Mixed Reality and Advanced Display, School of Optics and Photonics, Beijing Institute of Technology, Beijing 100081, China; State Key Laboratory for Mesoscopic Physics & Department of Physics, Collaborative Innovation Center of Quantum Matter, Beijing Academy of Quantum Information Sciences, Nano-optoelectronics Frontier Center of Ministry of Education, Peking University, Beijing 100871, China; Institute of Physics, The Chinese Academy of Sciences, Beijing 100191, China

**Keywords:** integrated photonics, inverse design, mode-division multiplexing, nanophotonics

## Abstract

Tunable mode converter is a key component of channel switching and routing for optical communication system by adopting mode-division multiplexing. Traditional mode converter hardly implements high-order mode conversion and dynamic tunability simultaneously. In this study, we design a tunable mode converter filled with liquid crystal, which can convert fundamental mode into multiple high-order modes (TE_0_, TE_1_, and TE_2_) with a good performance and low intrinsic loss. For this multiple-objective task, we propose an inverse design framework based on the adjoint method. To experimentally prove our design, a tunable mode converter filled with air or water and a mode demultiplexer are fabricated to implement dynamic routing. The experimental results agree well with the simulation and reveal the crosstalk only around −7 dB. With its performance and efficiency, our proposed design flow can be a powerful tool for multifunction device design.

## 1 Introduction

To meet the demand on the growing bandwidth of an optical communication system, various multiplexing technologies have been successfully developed and deployed, such as time-division multiplexing [1], wavelength-division multiplexing (WDM) [2], and polarization-division multiplexing [3]. But the problem of limited information capacity remains unsolved [4–6]. Recently, mode-division multiplexing (MDM) has attracted the attention of many researchers [7–9], due to its potential to expand information capacity and solve the bandwidth problem. In all-optical networks, the key component that enables the switching and routing in WDM is the optical cross-connect (OXC) [10]. The OXC consists of multiple wavelength-selective switches (WSSs), which can switch data among different light carriers with different channels [11]. Similarly, mode-selective switches are also key components in the MDM system. In general, a mode-selective switch can be realized by connecting a tunable mode converter (TMC) to a passive mode demultiplexer [12].

The mode converter can convert the fundamental mode into a selected high-order mode and vice versa. Different mode conversion devices have been designed and fabricated. For example, control over the mode can be achieved via the utilization of deep-subwavelength plasmonic waveguides in photonic-integrated circuit applications [13]. Alternatively, one can control the mode by coupling light into a wide slot photonic crystal waveguide [14]. TMCs based on tilted Bragg gratings and thermo-optic effect have a high coupling efficiency between the fundamental mode and different spatial modes [15]. However, limited by the thermo-optic effect, the switching speed of this kind of device is slow. Lithium niobate has a high electro-optic coefficient and modulation rate. However, it has a bulky structure due to the proton-exchange process for waveguides fabrication [16]. A programmable waveguide mode converter based on a phase-gradient meta-surface consisting of phase-change material Ge_2_Sb_2_Te_5_ (GST) can control the conversion of two spatial modes (TE_0_ and TE_1_ modes) precisely with 64 levels [17]. However, the intrinsic loss of this device is high due to the GST’s absorption in the crystalline phase, and the conversion to higher-order modes is not allowed.

The liquid crystal offers an excellent platform for electrical tunable multifunctional devices, due to its fast switching speeds, low loss, and mature processing technology. The challenge is designing multiple refractive-index states in a single geometry by the optimization algorithm. Heuristic optimization algorithms (e.g., genetic algorithms [18] or direct binary search (DBS) [19]) can search most of the parameter space, but the computation cost increase exponentially with increasing design parameters [20]. Recently, the deep learning method has been used to design nanophotonic devices, such as multi-mode interference (MMI) implementing arbitrary transmission matrices [21] and power splitters with arbitrary splitting ratios [22]. The size and effectiveness of the training dataset strongly determine the quality of the neural network [23]. However, the training dataset is usually obtained through full-wave simulations, which require a host of time and economic costs. Herein, we adopt an adjoint method to address the optimization task of multi-target functions. Compared to other design methods, the adjoint method can obtain the gradient of an objective function with respect to all design degrees of freedom through two full-field simulations [24]. This optimization method is suitable for a structure that simultaneously achieves various design objectives and has a very large parameter space, such as an on-chip three-channel wavelength demultiplexer [25], broadband polarization beam splitter [26], color and polarization filter [27], and large-scale metasurface [28, 29].

We can scale this device’s function by adding the degree of freedom or enlarging the refractive index of tunable material. This scalability comes from the adjoint method’s performance and the liquid crystal tunability. Compared with the reconfigurable mode converter based on subwavelength Y-junctions [30], our device has a smaller footprint and faster switching speed. However, the scalability of our device is a little bit weaker than the counterpart based on subwavelength Y-junctions, and this is limited by the design method we used. A better way to improve the scalability is to combine our LC device with the subwavelength Y-junctions. Polarization control is one of the important issues in photonic integrated circuits. Mode and polarization-division multiplexing (MDM and PDM) can offer considerable parallelism for high-capacity optical interconnect, while requiring only a single-wavelength laser source [31]. Meanwhile, the wavelength-division multiplexing (WDN) technique is one of the most popular technologies and has been developed very successfully in past decades. The introduction of MDM in the WDM systems can greatly reduce the number of arrayed-waveguide gratings (AWGs) under the same information capacity, thereby reducing the footprint [32]. In summary, due to the compact structure of our device, it can integrate into a variety of systems to expand information capacity, such as WDM systems.

**Figure 1:**
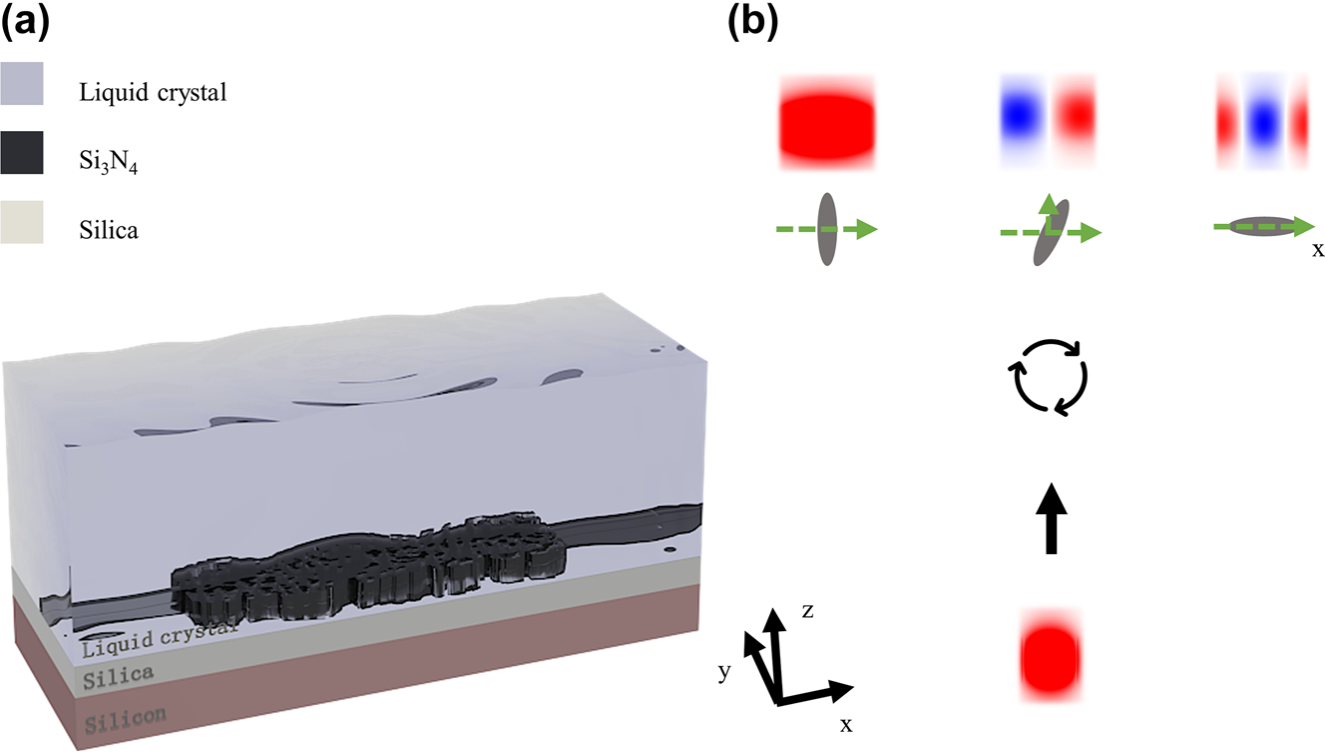
Schematic of the TMC. (a) Schematic of the TMC covered by LC, the TE_0_ to TE_0_ (TE_0_ to TE_1_, TE_0_ to TE_2_) mode converter with the 1 μm wide input waveguide to the 2 μm wide output waveguide. (b) The incident TE_0_ mode can be converted to the TE_2_ mode when the LC director is aligned along the *x*-axis, converted to the TE_1_ mode when the LC director is rotated 33° counterclockwise and converted to the TE_0_ mode when the LC director is perpendicular to the *x*-axis.

In this work, a tunable mode convertor has been realized by inverse design method based on adjoint simulation and verified experimentally. We simulate an MMI with a patterned coupler region covered by LC to convert the fundamental mode into a higher-order mode (TE_0_, TE_1_, or TE_2_) on an 800 nm Si_3_N_4_/3.3 μm SiO_2_ platform [Figure 1(a) and (b)]. In order to experimentally validate the feasibility of this device, we design and fabricate a tunable counterpart, which can convert TE_0_ into TE_1_ by replacing the environment of water to air on a 220 nm thick top silicon-on-insulator (SOI). We also design a mode-demultiplexer to realize an optical routing combined with this TMC. The pixel size is chosen to be 50 nm × 50 nm and the restriction methods are employed for this density-based topology optimization, which provides a good trade-off between fabrication accuracy and degree of design freedom. Apart from Gaussian-blurring, binary morphology operators are used to enforce strict minimum feature size constraints. The morphology operators can remove all objects or holes with dimensions smaller than the minimum feature size, and it hardly affects the performance of the resulting device. Removing the pillars and holes with a big depth-to-width ratio can increase system robustness and weaken the disturbance of the liquid crystal inhomogeneities. The minimum feature size of the optimized device is 100 nm, which is suitable for conventional nano fabrication techniques such as electron-beam lithography (EBL) and focused ion beam (FIB). In 3D full-wave simulation, this mode converter, with a footprint of 4 μm × 9 μm, exhibits an intrinsic loss of 0.5 dB (TE_0_ to TE_1_) and 0.7 dB (TE_0_ to TE_0_) at 1550 nm, and the experimental measurements are in good agreement with the simulations. The optimization framework we proposed offers a path to high-efficiency tunable devices as a key component of the optical communication system based on MDM.

## 2 Methods

The device of the control mode is designed by the adjoint technique, wherein the pattern of the design area is updated in every iteration to maximize the figure of merit (*FOM*).

**Figure 2:**
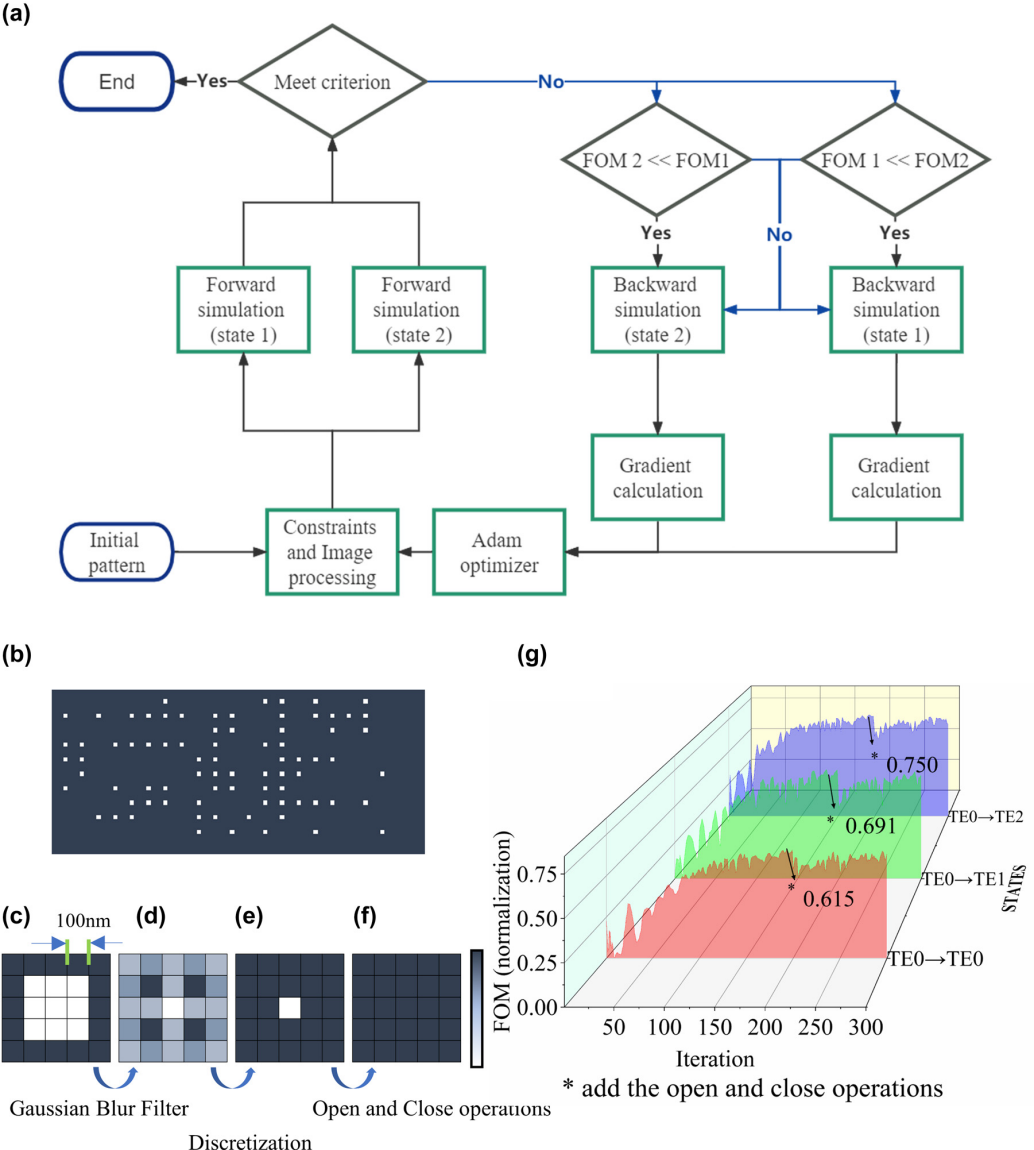
The flow chart of the inverse design method. (a) The flow chart of the inverse design method. (b) The initial pattern of topology optimization. The dark blue blocks represent cells made of silicon nitride. (c) Partially enlarged view of the design region. (d) Structure after Gaussian blur filter. (e) Structure after discretization. (f) Structure after open and close operations. (g) Evolutions of *FOM* over the topology optimization process.

The flow chart is shown in Figure 2(a). We first randomly initialize the design parameters and then enforce the constraints and image processing to make the optimized design follow the design rule checking (DRC) for the fabrication process. The obtained density distribution was imported into COMSOL and then interpolated as the relative permittivity by the nearest neighbor method. The *FOM* and electric field distribution of the design area can be obtained when setting an excitation power at the input port (forward simulation). We add two decisions before the backward simulation to penalize the state that converges the fastest. This operation not only reduces running time but also improves the average performance of the multi-state device (Supplementary note 3). The gradient can be obtained from the electric field of the forward and backward simulation. Finally, we use the adaptive moment estimation (Adam) to process the gradient, and it can help the device converge to an optimal solution. In general, the Adam optimizer is performance in stochastic optimization. In Supplementary note 2, we compare the performance of the SGD (Stochastic Gradient Descent) and the optimization framework with logic branching.

### 2.1 Initial pattern

The initial value of the TMC filled with LC is shown in Figure 2(b). In the coupling region, one hundred square pixels were randomly distributed in the 20 × 10 square grid. The size of each pixel is 100 nm × 100 nm. For such an initial value, its optimized structure can maintain integrity, in another word, the resulting device is mechanically stable. Furthermore, compared to the uniform initial distribution (the relative dielectric constant is midway between those of the two materials), such an initial value has the same rate of convergence.

### 2.2 Constraints and image processing

The LC is a birefringent material with an ordinary (*n*_*o*_ = 1.5002) and extraordinary (*n*_*e*_ = 1.6868) refractive index and an optic axis that coincides with the director. The *c*-axis of LC lies in the XY plane and makes an angle of *θ* with the *x*-axis, and its relative permittivity tensor is defined as

(1) $$\begin{array}{l} \begin{array}{cc} \varepsilon_{LC} & =\left[\begin{array}{ccc} \varepsilon_{xx} & \varepsilon_{xy} & \varepsilon_{xz}\\ \varepsilon_{yx} & \varepsilon_{yy} & \varepsilon_{yz}\\ \varepsilon_{zx} & \varepsilon_{zy} & \varepsilon_{zz} \end{array}\right]=\left[R\right]\cdot \left[\varepsilon \right]\cdot {\left[R\right]}^{T}\\ & =\left[\begin{array}{ccc} \cos\left(\theta \right) & -\sin\left(\theta \right) & \\ \sin\left(\theta \right) & \cos\left(\theta \right) & \\ & & 1 \end{array}\right]\left[\begin{array}{ccc} {n_{e}}^{2} & & \\ & {n_{o}}^{2} & \\ & & {n_{o}}^{2} \end{array}\right]\\ & \;\times \left[\begin{array}{ccc} \cos\left(\theta \right) & \sin\left(\theta \right) & \\ -\sin\left(\theta \right) & \cos\left(\theta \right) & \\ & & 1 \end{array}\right] \end{array} \end{array}$$

where *R* represents the rotation matrix. For the implementation of LC devices, the anchoring effect is an import problem, and it will greatly deteriorate the switching speed and performance of the device. There are several ways to solve these problems such as calculating refractive index distribution using free energy minimization of a Landau–de Gennes continuum modal [33], reducing the contribution of the substrate to the metamaterial surface area [34]. For this density-based topology optimization, the density (*ρ*) is linearly interpolated to yield the permittivity of the design area [24]. And the relative permittivity of the silicon nitride equals 3.984 at 1550 nm.

In the optimization process, the pattern of the design area would include small features that are not manufacturable. To ensure the design can be transferred to a manufacturable geometry, we apply a Gaussian filter to the pattern to avoid small features [Figure 2(d)]. Here, we choose a filter radius of *R* = 200 nm. In addition, the optimization results often include intermediate permittivity values. A projection method is employed to obtain binary solutions in the optimization [Figure 2(e)] [35].

The Gaussian filter was often used in the inverse design, but there is a blemish for the multi-function optimization. That is, although the Gaussian blur can remove small features and maintain the pattern’s structural integrity, it severely limits design degrees of freedom when the filter radius is slightly increased so, we reduce the radius and add the morphology operators (“open” and “close” operations). The open and close operations can enforce the constraints barely affecting the resulted device’s performance, though it doesn’t make the pattern integrity. The basic morphology operators are “erode” and “dilate,” and several extensions can be performed by sequentially applying the erode and dilate operators. Take the erode operation as an example, if any of the pixels covered by the neighborhood is void, then the center pixel is made void. The “open” and “close” operations we used are defined as an erode followed by a dilate operation and a dilate followed by an erode operation, respectively. The open operation can remove all objects with dimensions smaller than the filter size, while the close operation can fill in holes with dimensions smaller than the filter size [Figure 2(f)] [36]. This process can be defined as,

(2) $${{\bar{\tilde{\rho }}}_{i}}^{O/C}=O\left(C\left(\bar{\tilde{\rho }}\right)\right)$$

where *O* represents the open operator and *C* represents the close operation.

### 2.3 FOM

The device is designed to control the conversion of the waveguide’s multiple spatial modes through the LC director change, and our optimization process is aimed at a single wavelength. The optimization of nanophotonic devices is viewed as a maximization problem of the desired design objective function *FOM*, the objective function can be formulated as,

(3) $$\begin{array}{c} \underset{{\tilde{\bar{\rho }}}^{O/C}}{\max}FOM\left(E\right)=\sum \begin{array}{c} N\\ s=1 \end{array}\frac{\left\langle E^{s}\left|\right.\overset{s}{\underset{\text{target}}{E}}\right\rangle }{\left\langle \overset{s}{\underset{\text{target}}{E}}\left|\right.\overset{s}{\underset{\text{target}}{E}}\right\rangle }\cdot \omega_{s}\\ \sum \omega_{s}=1 \end{array}$$

where ***E*** represents the electrical field profile of output waveguide, $E_{\text{target}}^{s}$ represents the desired electrical field profile of output waveguide, and superscript *s* represents the *s*th state of a multi-state task. Our optimization process is aimed at a single wavelength. In the revised manuscript, we have added the above explanation.

### 2.4 Forward simulation and backward simulation

We solved the optimization problem given by

(4) $$\begin{array}{c} \underset{E^{1},E^{2},E^{3},{\tilde{\bar{\rho }}}^{O/C}}{\max}FOM\left(E^{s}\right)\\ subject\;\;to\nabla \times \frac{1}{\mu_{0}}\nabla \times E^{s}-\omega^{2}\varepsilon \left({\tilde{\bar{\rho }}}^{O/C}\right)E^{s}=-i\omega J^{s}\\ s=1,2,3... \end{array}$$

where *E*^*s*^ is the electric field at the *s*th state, *J*^*s*^ is the current source to excite the TE mode of the input waveguide, and *ρ* parametrizes the structure. To solve for the derivative of total *FOM* with respect to *ρ*_*i*_, we need to calculate the forward and adjoint electric field using the full-wave simulation. The adjoint field can be considered as the field that is produced by injecting fields that get from ∂*FOM*/∂ ***E*** at the output port [24]. The gradient calculated from the electromagnetic fields obtained by two COMSOL simulations can be expressed as:

(5) $$\frac{dFOM}{d\rho }=2Re\left\{{\overset{⃑}{E}}_{\text{fwd}}\cdot {\overset{⃑}{E}}_{\text{adj}}\right\}$$

Finally, *ρ* is updated using the Adam algorithm, which is a variant of gradient descent.

The structure is variant in the *z*-axis due to the covering layer of LC in the device, so the 2D simulation will converge to the inaccuracy solution compared to the real device. For making a trade-off between the computational cost and error with the experimental results, we take the results from 2D simulation as the initial value of 3D simulation. In Figure 2(g), we have plotted the *FOM*s as a function of the number of iterations in 3D simulation. During the earlier stage of the optimization process, the average *FOM* improves from 0.1 to 0.504 (at the 70th iteration) rapidly. This fast convergence rate comes from the fact that this algorithm can compute gradients to update the structure. Note that the *FOM*s have a slight drop at the 200th iteration because we add the open and close operations. At 292nd iterations, the average *FOM* can reach 0.685, showing almost the same value as before adding the morphology operators. It proves that this algorithm framework can design efficiently CMOS compatible, multifunctional, structural integrity, and high-performance devices.

## 3 Results

**Figure 3:**
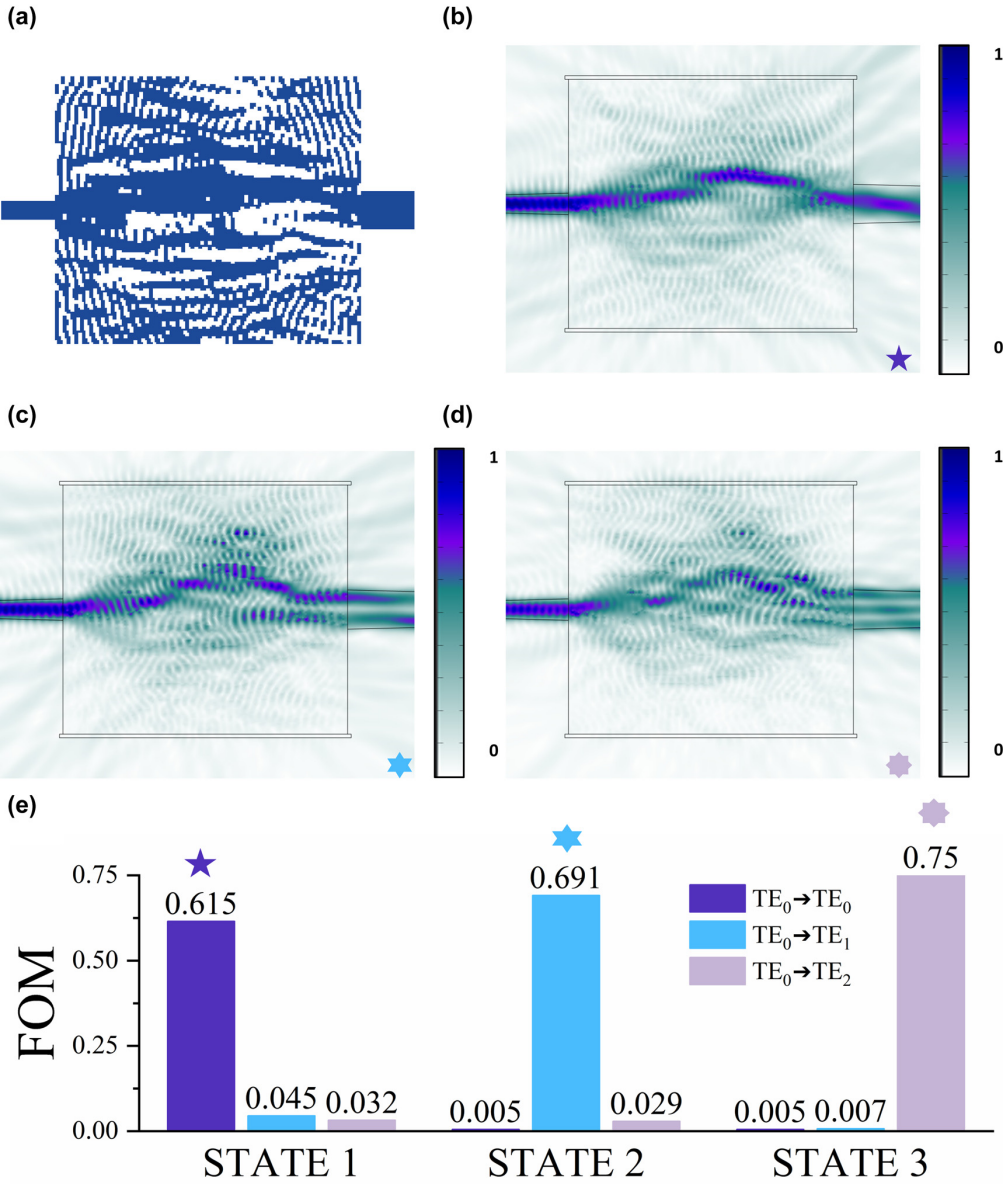
Simulation results of the tunable mode converter covered by LC. (a) The optimized density distribution of the TMC, input waveguide, and output waveguide. (b) In state 1, the light field distribution. (c) In state 2, the light field distribution. (d) In state 3, the light field distribution. (e) The *FOM* of three states. Our desired output mode’s light intensity is much higher than the intensity of the other mode.

We use the optimization framework to inverse design TMC covered by LC on an 800 nm Si_**3**_N_**4**_ platform. For the LC material, we use E7 [37], which has a refractive-index variation Δ*n* of about 0.1855 between the voltage-on and voltage-off states. This E7 liquid crystal with a large birefringence was chosen because we prove that the birefringence is correlated with the performance of the optimized device (Supplementary note 4). This device can convert the fundamental mode into a higher-order mode (TE_0_, TE_1_, and TE_2_) according to the angle between the optic axis and the *x*-axis. Here, we set state 1 as *θ* = 0°, state 2 as *θ* = 33°, and state 3 as *θ* = 90°. As shown in Figure 3(a), the designed mode converter is composed of a 1 μm wide input waveguide, a 2 μm wide output waveguide, and a coupling region of 14 μm × 16 μm. The light intensity distributions at the middle of the 800 nm silicon nitride layer are simulated by 3D COMSOL shown in Figure 3(b)–(d). At different states, the input fundamental mode is converted to TE_0_, TE_1_, and TE_2_ mode accordingly. The optimized design exhibits an extraordinary performance at 1550 nm wavelength. As shown in Figure 3(e), the purity of the desired mode of output port at different states is very high, and the transmitted energy of the other mode is less than one percent of the desired mode.

**Figure 4:**
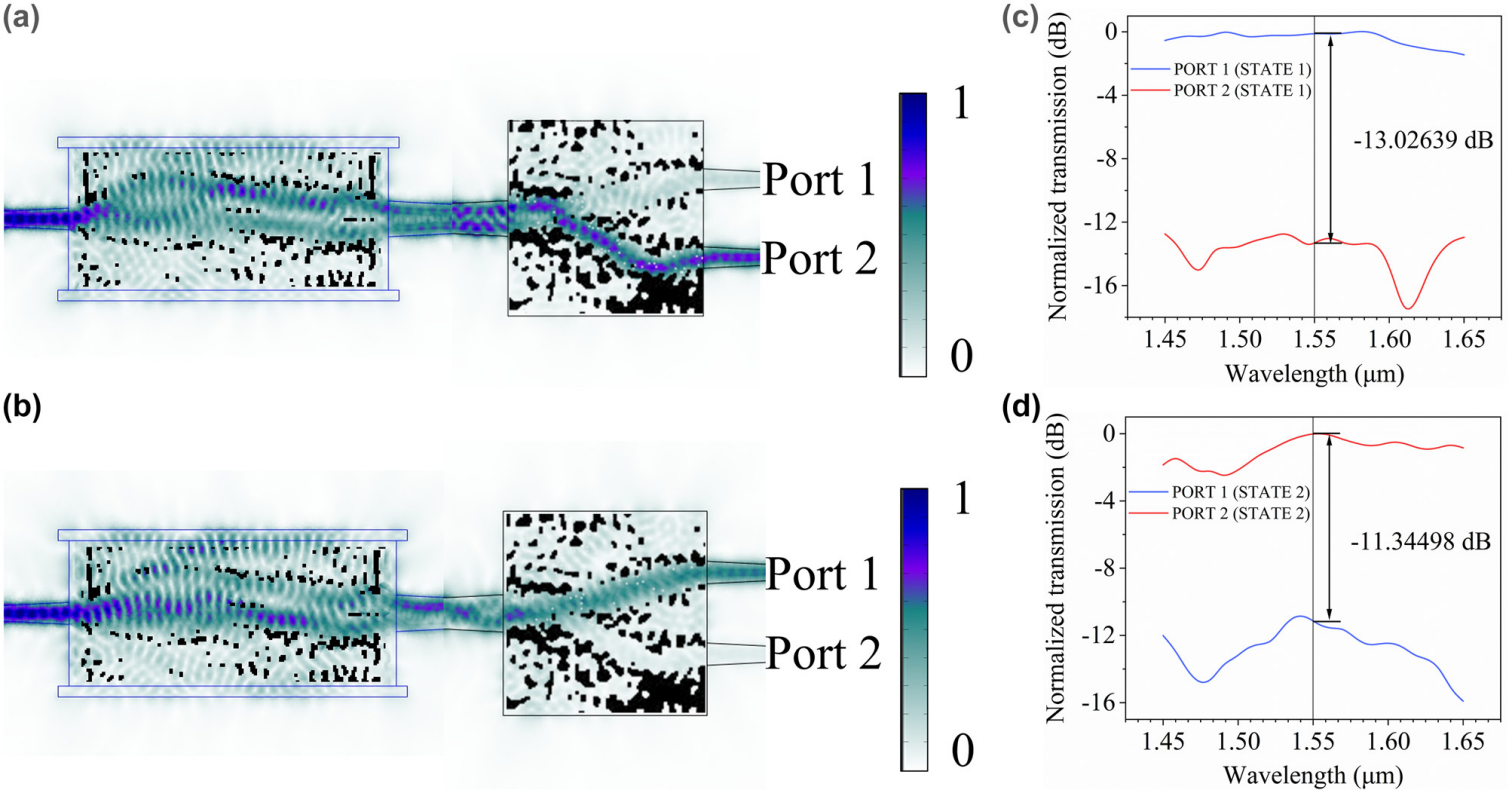
Simulation results at different states. (a) and (b) The light intensity distribution of TMC and mode demultiplexer filled with H_2_O and AIR, respectively. (c) and (d) The simulated spectral responses of normalization transmission for input TE_0_ and TE_1_, respectively.

In addition, we design and fabricate a tunable counterpart filled with air or water to experimentally validate the feasibility of LC devices because of the difficulty of packaging and manufacturing liquid crystal devices. Although the covering medium of the fabricated device is not LC, the design flow and physical mechanisms of our inverse design on the tunable on-chip device are the same. The change of background refractive index between water and air can mimic the refractive change of LC. This device is fabricated on an SOI wafer with a 220 nm thick top silicon layer and a 3 μm thick buried silicon dioxide layer, which can convert TE_0_ into TE_1_ or not. The pattern of this system was etched by focused ion beam. For observing the mode conversion obviously, we design a mode-demultiplexer with a footprint of only 5.5 μm × 5.5 μm to switch output ports based on the mode of the input port [Figure 4(a) and (b)] by our inverse design framework with logic branching. And in the experiment, this is difficult to only drip on the mode converter and ensure that water will not volatilize before the experiment is completed. The change of this refractive index is considered in the design of the mode demultiplexer. The crosstalk of mode-demultiplexer reaches −13.026 dB and −11.345 dB for input TE0 and TE_1_ at 1550 nm [Figure 4(c) and (d)] through 200 iterations, and this device can maintain a good performance within the whole C-band simulated by 3D COMSOL. And no matter in water or air, the mode demultiplexer can have small crosstalk (Supplementary note 5). We also did the back-to-back test of this device (Supplementary note 6). Compared with other mode division demultiplexers [38, 39], this mode demultiplexer has a broadband spectrum response and a small footprint, and it is very suitable for integration with other on-chip optical devices. The black blocks in [Figure 4(a) and (b)] are optimized patterns etched fully on a 220 nm top silicon-on-insulator. Focused ion beam is used to etch this pattern and waveguides. In focus ion beam, the round corner effect is one of the typical etching errors. We study the impact of variations of rounding radius (Supplementary S1).

**Figure 5:**
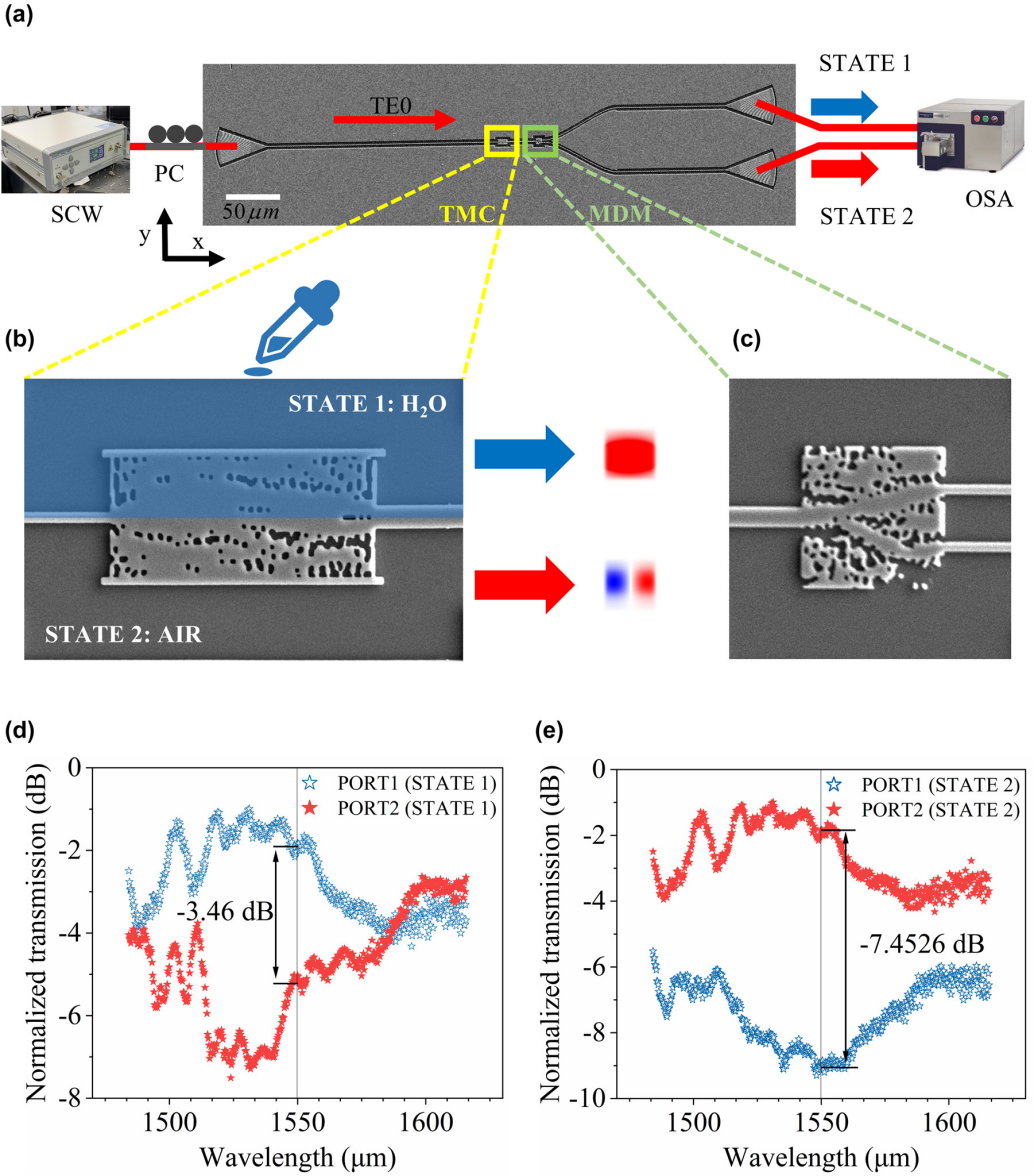
The experimental setup and the scanning electron microscopy image of our system. (a) Experimental setup for device characterization (SCW, supercontinuum wave laser; PC, polarization controller; TMC, tunable mode converter; MDM, mode demultiplexer; OSA, optical spectrum analyzer). The top-down view of the device is taken by scanning electron microscope. The yellow box indicates the tunable mode converter, and the green box indicates the mode demultiplexer. (b) The top-down view of the mode converter can implement tunable mode conversion by dropping a small amount of water on this device. (c) SEM image of the mode demultiplexer. (d) and (e) The experimental results for the whole system. Measured transmission spectra of the two output ports, validating the functionality of these devices.

The scanning electron microscope (SEM) of this system is shown in Figure 5(a). The optical signal is generated by a supercontinuum wave laser (SC-5, YSL), coupled into the system by grating couplers. The test details are as follows. First, we adjust the polarization controller and ensure that the polarization direction of the light received by the input port is parallel to the *Y*-axis. Second, adjust the position and height of optical fiber (the fiber ends are cut) to maximize the coupling efficiency of grating and fiber under optical microscopes. The input mode can be converted into TE_1_ mode through the TMC when we don’t drop water in the design area [Figure 5(b)]. Then the mode demultiplexer can split TE_0_ mode and TE_1_ mode from an input multi-mode waveguide into one of the two output single-mode waveguides, and the SEM image of this device is shown in Figure 5(c). Finally, the optical spectral response of this system can be measured by the optical spectrum analyzer (model Andor 303i).

The transmission measured through each device was normalized, and this eliminated coupling and waveguide loss to yield a direct measurement of the mode converter and mode demultiplexer efficiencies. The measured results are shown in Figure 5(d) and (e), Crosstalk is −3.4600 dB and −7.4526 dB in two states at 1550 nm. As shown in Figure S1, the mode converter is a narrow-band device, and the peak of FOM is not at the 1550 nm we designed. Figure 4(c) and (d) indicate that MDM is a broadband device in the C band. In summary, the experimental data should have a good performance in a narrow band, and the peak of performance is not at 1550 nm. And in Figure 5(e), there is a slight decrease in crosstalk at other wavelengths. It can be seen that the experimental and theoretical line shapes are mostly consistent. We attribute the discrepancy between simulation and measurement to fabrication imperfections. During fabrication, the faint fluctuations of the ion beam dose and wafer surface’s roughness are not considered, which will lead to decreased efficiency and the stability of the mode. In the next work, we will optimize the fabrication process to reduce errors. When we drop a little water on the chip, the loss of coupling between the optical fiber and grating and the loss of the device will increase to deteriorate crosstalk.

## 4 Conclusion

In this study, we developed an adjoint method with multiple constraints, and this algorithm can be applied to any multi-state optical device. These constraints were incorporated in a fully automated inverse design method in the form of a set function. We made a trade-off between the conversion efficiency and fabrication accuracy by reducing the filter radius of Gaussian blur and adding morphology operators. Using this algorithm, we optimized a tunable mode converter with a compact structure. The mode converter covered by LC can convert fundamental mode into higher-order mode according to the arrangement direction of LC molecules. The designed device exhibits a great performance at 1550 nm with a footprint of only 14 μm × 16 μm. In order to demonstrate the feasibility of this kind of device, we design and fabricate a tunable counterpart covered by air or water on a 220 nm SOI platform. The change of different covering mediums can mimic the refractive index change of LC. We also design and fabricate a mode demultiplexer to realize optical routing. The crosstalk is −3.46 dB and −7.45 dB in two states at 1550 nm, so the TMC can implement the mode conversion greatly.

In summary, we have experimentally demonstrated a compact, practical mode converter and demultiplexer designed using our inverse design algorithm. To improve optimization with large minimum feature sizes and a wide range of wavelengths, future work might focus on optimizing initial conditions and combining the application of adjoint-based local-optimization techniques within a larger global-optimization platform. We can use this approach to discover a multi-state device with high efficiency and compactness. Our results reveal a promising component for flexible dense MDM systems, as a key component of optical cross-connect and routers.

## Supplementary Material

Supplementary Material Details
