# Supplementary material for: Tunable on-chip mode converter enabled by inverse design
Source: Nanophotonics. 2023 Feb 17;12(6):1105–14. doi: 10.1515/nanoph-2022-0638 (PMC11502087; doi:10.1515/nanoph-2022-0638)
Supplement: Supplementary file 1 — Supplementary Material Details [file j_nanoph-2022-0638_suppl.docx]

**Supplementary Information for**

**Tunable on-chip mode converter enabled by inverse design**

**Supplementary Note 1: The analysis of fabrication tolerance for mode converter**

In focused ion beam, the round corner effect is one of the typical etching errors. The blue box in Figure S1(a) indicates a nonzero rounding radius at both the inner and outer corners. We study the impact of variations of rounding radius (0 nm, 5 nm, …, 20 nm), which are described in [Figs. 1(a) and 1(b)]. We can see that the fabrication error of the round corner effect has little influence on the performance, shown in the insets of [Figs. 1(a) and 1(b)].

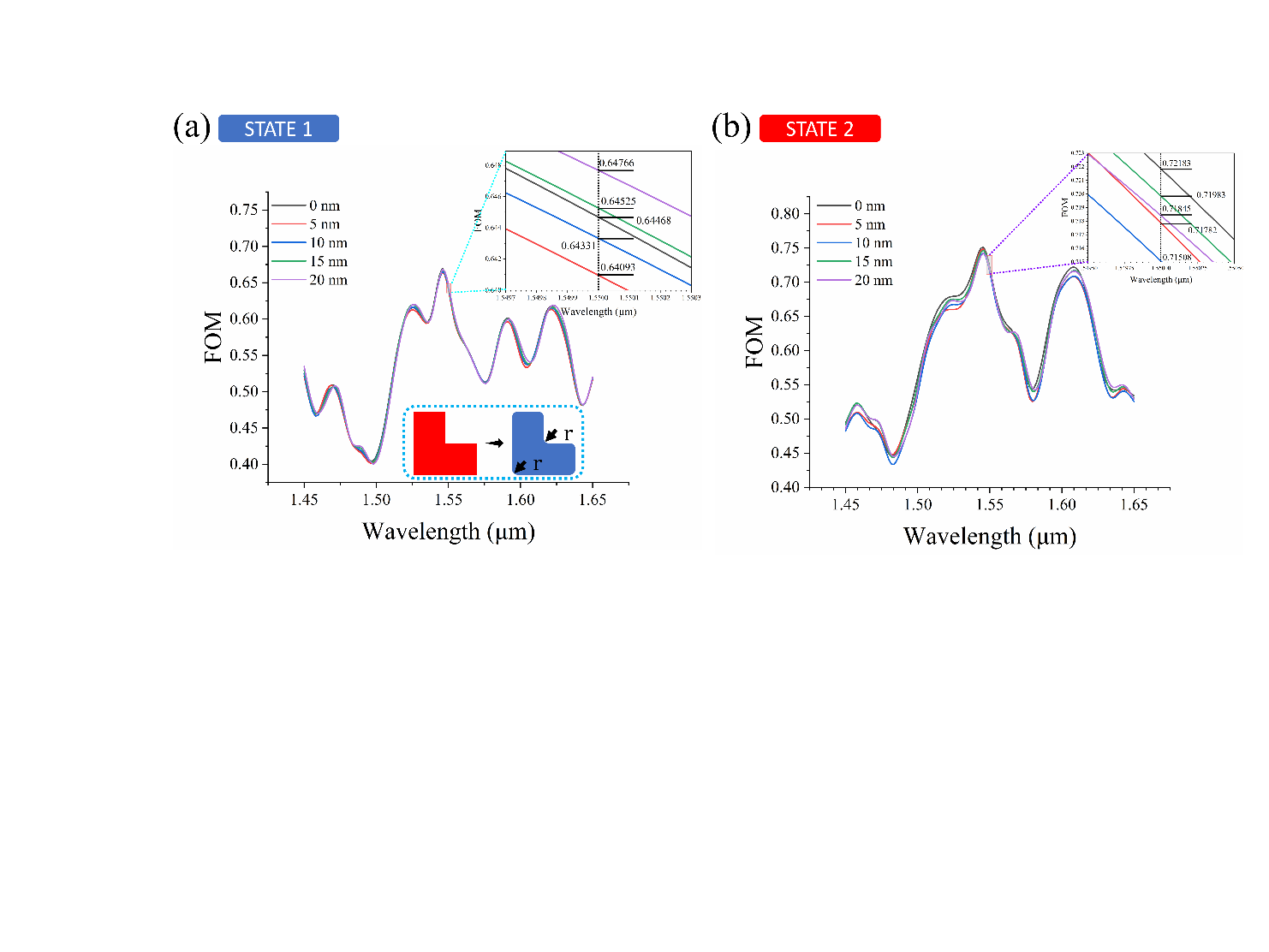


Figure S1. The analysis of fabrication tolerance for mode converter. (a, b) The simulated spectral responses consider the fabrication tolerance of different rounding radii. The blue box indicates the sharp corner and rounding radius of the rectangle.

**Supplementary Note 2: results through SGD (Stochastic Gradient Descent)**

The ADAM optimizer is a performance for stochastic optimization, and this is an accepted fact in many fields. However, for our multi-target task, its performance is worse than its counterpart with logic branching. As shown in Figure S2, the results through the SGD optimization are not as good as expected, and it is not even better than the results obtained through logical branching. The evolution of FOM has a very large fluctuation (Figure S3), although we have chosen the appropriate learning rate and adjusted other parameters. And FOMs of the final device is degraded after using SGD.


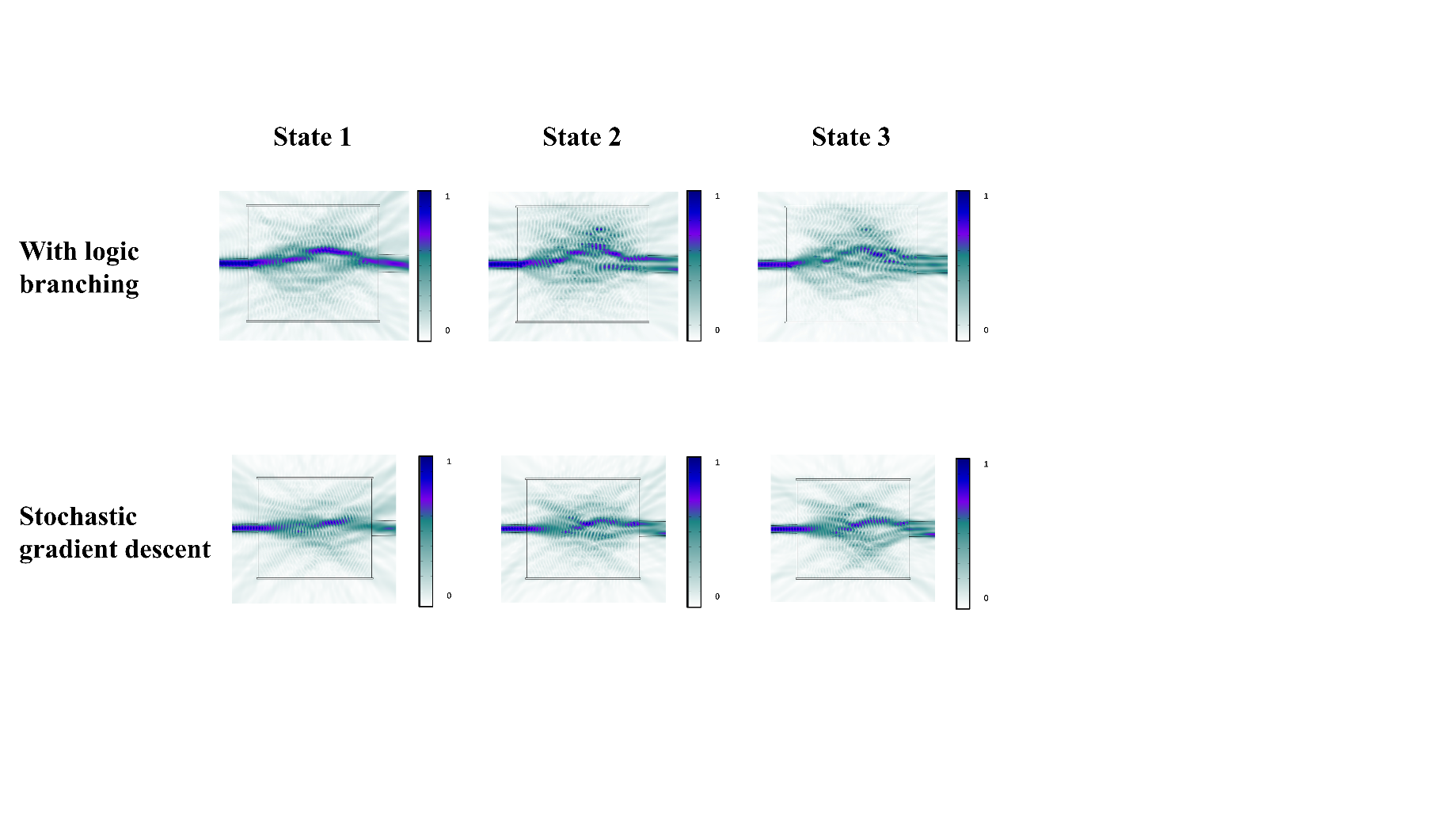


Figure S2 The optimized device’s light distribution through COMSOL. The row number represents the optimization method, which is the ADAM optimizer combined logic branching and SGD; The column represents the light distribution of the device in different LC states.





Figure S3 Evolutions of FOM over the SGD.

**Supplementary Note 3: verifying the effectiveness of adding logic branching**

When we don’t add logic branching (Figure S4(b)), the device in state 1 will quickly converge to a large value. This situation seriously affects the performance of other states. Compared with Figure S4(a), the above results prove that the logical branching we proposed can improve the average performance. About how to reduce the running time, this is actually to shorten the running time by reducing the number of iterations.


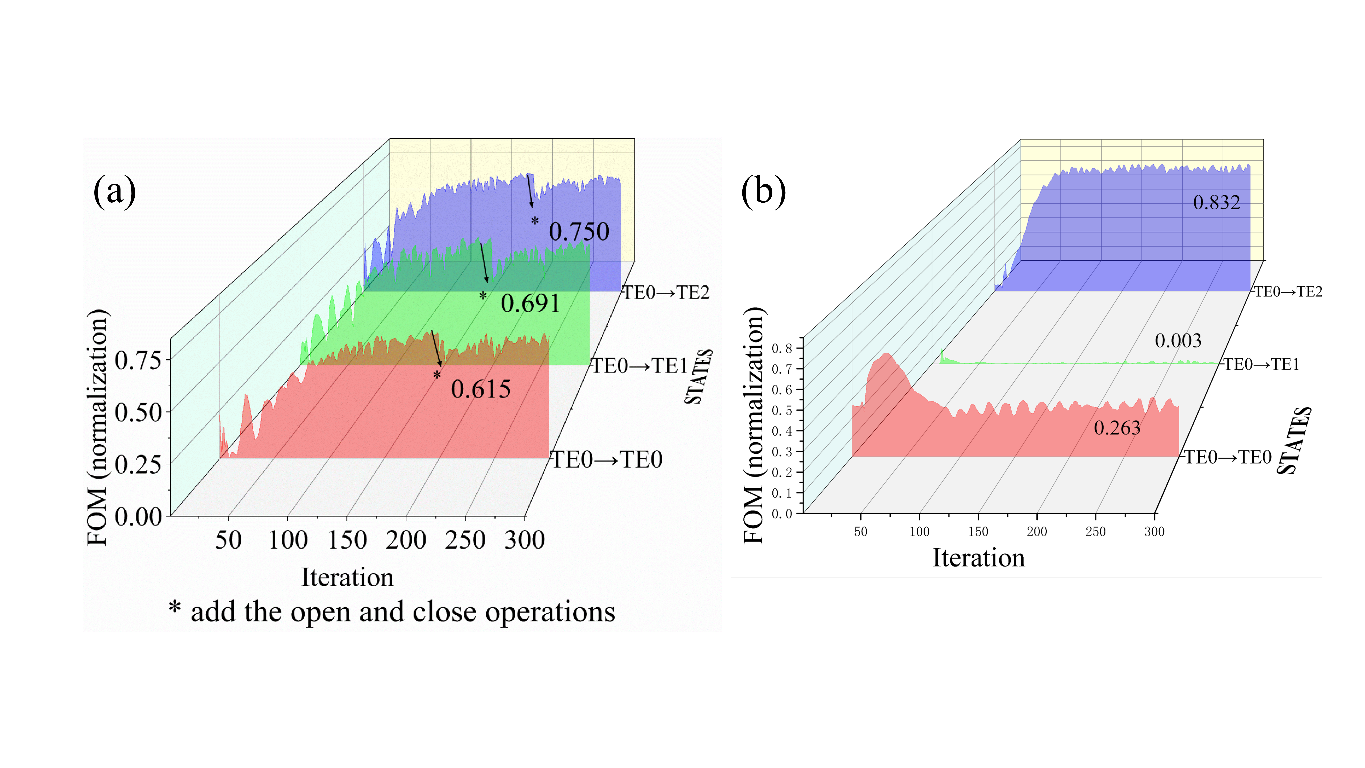


Figure S4 Evolutions of FOM (a) added logic branching. (b) not added logic branching.

**Supplementary Note 4: the relationship between birefringence of the LC and final device performance**

We use three liquid crystals to explore FOM and birefringence relationships through COMSOL. The refractive index data of the three LCs are shown in Table 1. These refractive index data are imported into our design framework under the same parameters, and then we obtain the relation between FOM and birefringence. (Figure S5) And we see that the greater the birefringence of the material, the better the performance of the optimized device.

Table 1 Fitting refractive indices(ne and no) and birefringence Δn of MLC-9200-100, 5CB, E7 through Cauchy equation at 1550nm

| Materials | n_e_ | n_o_ | Δn |
| --- | --- | --- | --- |
| MLC-9200-100 | 1.5766 | 1.4766 | 0.1000 |
| 5CB | 1.6746 | 1.5162 | 0.1584 |
| E7 | 1.6838 | 1.5022 | 0.1816 |





Figure S5 The FOM of three states using three LCs.

**Supplementary Note 5: the impact of background refractive index on MDM crosstalk**

Indeed, this is difficult to only drip on the mode converter and ensure that water will not volatilize before the experiment is completed. The change of this refractive index is considered in the design of the mode demultiplexer. The designed mode demultiplexer can work whether the whole device is in the water environment or in the air. No matter whether in water or air, the mode demultiplexer can have small crosstalk (Figure S6(a-d)). Figures S6(a) and S6(d) show that if the TMC completes the mode conversion perfectly, the normalization transmission was obtained through entered corresponding mode light into the MDM. In fact, the TMC will generate some other mode that we don’t expect, which will reduce the crosstalk of the system (Figures S6(b) and S6(c)).


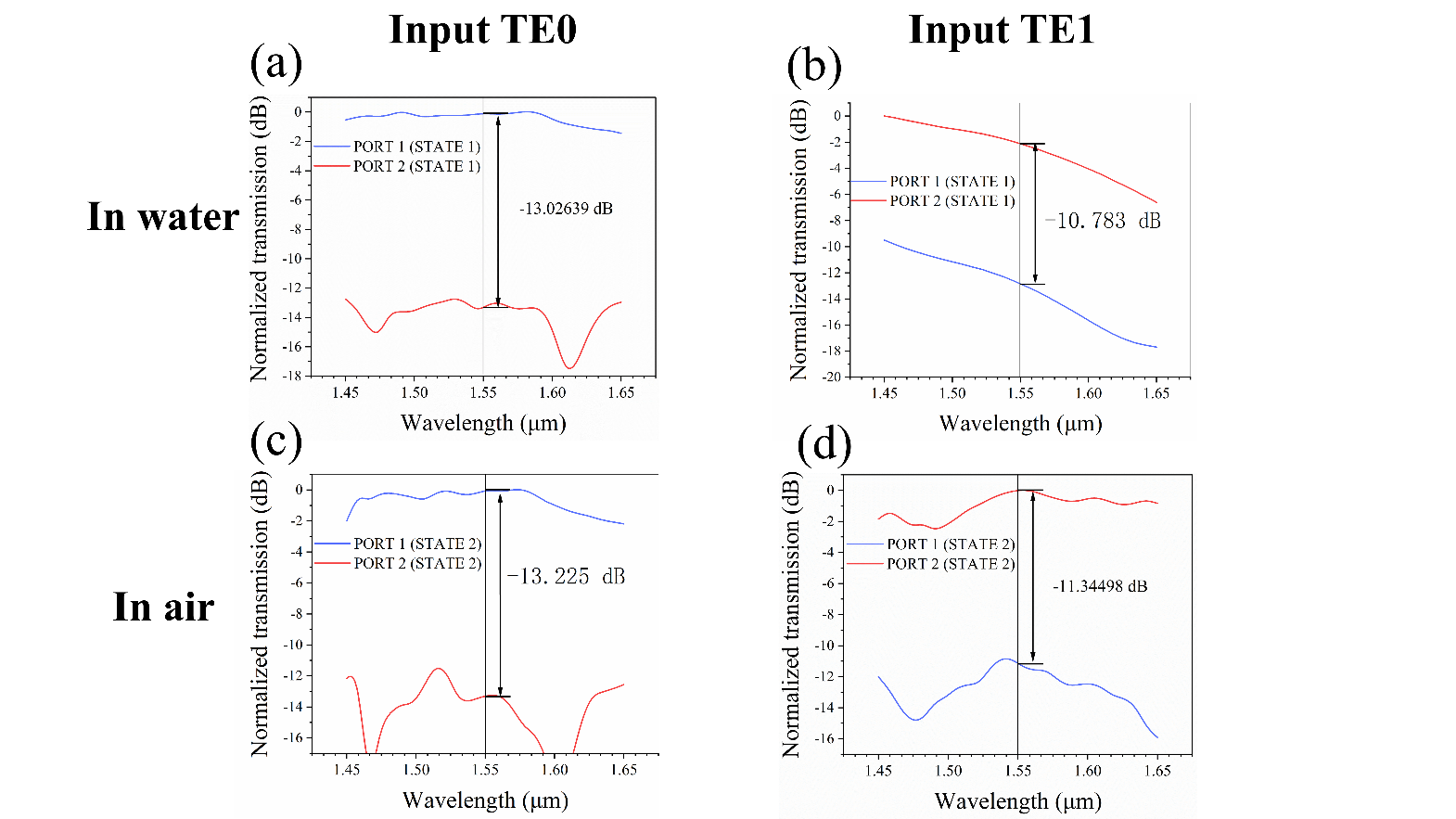


Figure S6 (a-d) the simulated spectral responses (MDM) of normalization transmission for input TE0 or TE1 and in air or water, respectively.

**Supplementary Note 6: the back-to-back test of the MDM demultiplexer**

In Figure S7, when the device is in water, the result of the third column is better than the result of the fourth column. Since the MDM demultiplexer is directly connected to the TMC, we do not need to consider the input of TE1 mode light in water. The similar thing happens when the device is in air.


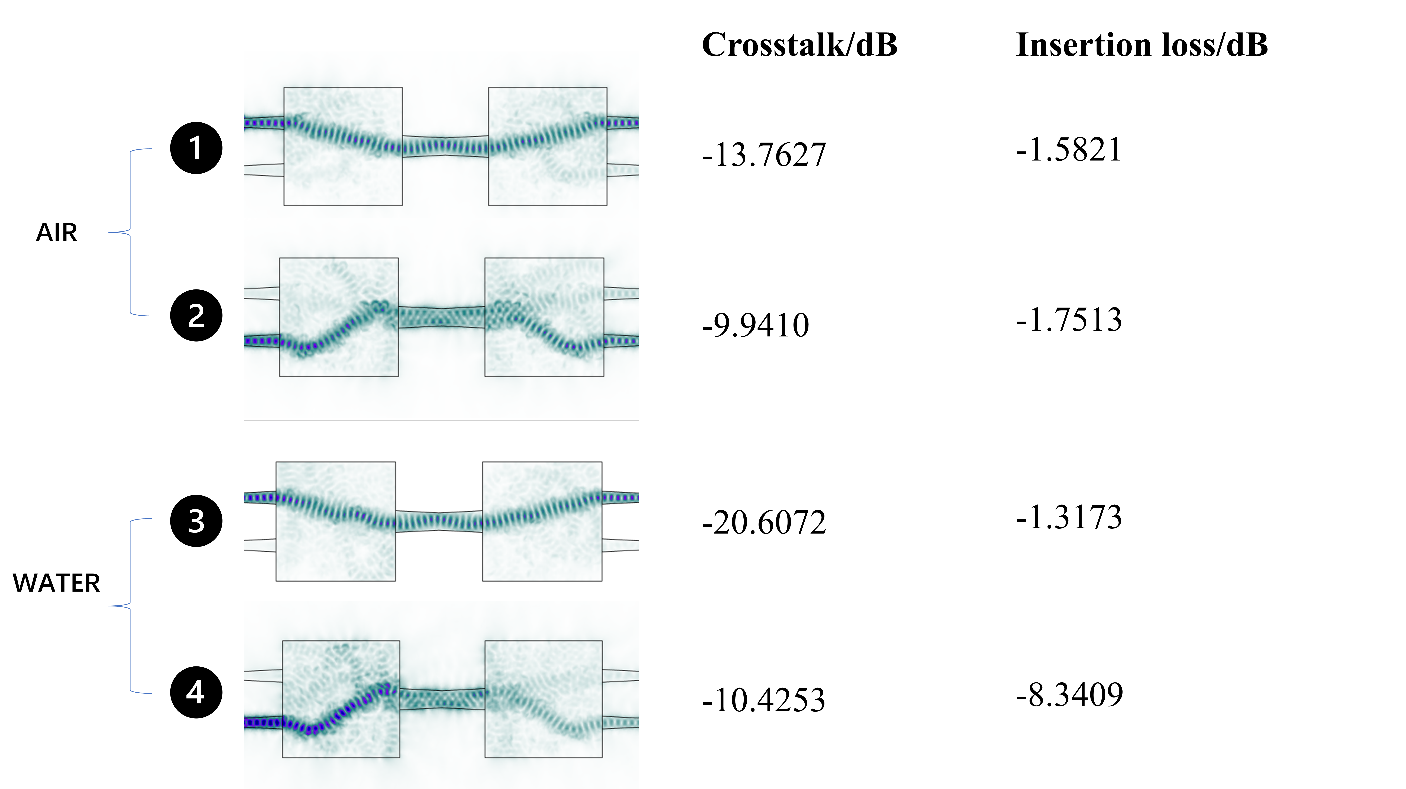


Figure S7 the light intensity distribution, crosstalk, and insertion loss of the back-to-back MDM demultiplexer.
